# Molecular Profiling and Survival Outcomes in Pancreatic Ductal Adenocarcinoma: A Multicenter Real-World Study from Turkey

**DOI:** 10.3390/curroncol33040216

**Published:** 2026-04-15

**Authors:** Selami Bayram, Bahadır Köylü, Maral Martin Mıldanoğlu, Mustafa Serkan Alemdar, Tahir Yerlikaya, Fatih Selçukbiricik, Ahmet Bilici, Ali Murat Tatli, Mustafa Ozdogan

**Affiliations:** 1 Department of Medical Oncology, Memorial Antalya Hospital, 07025 Antalya, Turkey; 2 Department of Internal Medicine, Division of Medical Oncology, School of Medicine, Koç University, 34450 Istanbul, Turkey; bkoylu@ku.edu.tr (B.K.); fselcukbiricik@ku.edu.tr (F.S.); 3 Department of Medical Oncology, Faculty of Medicine, Istanbul Medipol University, 34810 Istanbul, Turkey; maral.mildanoglu@medipol.edu.tr (M.M.M.); ahmet.bilici@medipol.edu.tr (A.B.); murat.tatli@memorial.com.tr (A.M.T.); 4 Department of Medical Oncology, Medikal Park Antalya Hospital, 07160 Antalya, Turkey; msalemdar@gmail.com; 5 Department of Medical Oncology, Antalya State Hospital, 07070 Antalya, Turkey; drtahiryerlikaya@gmail.com; 6 Department of Medical Oncology, Memorial Göztepe Hospital, 34634 Istanbul, Turkey; mustafa.ozdogan@memorial.com.tr

**Keywords:** pancreatic ductal adenocarcinoma, next-generation sequencing, KRAS, TP53, tumor mutational burden, molecular profiling, overall survival, progression-free survival, precision oncology, real-world study

## Abstract

**Background:** Pancreatic ductal adenocarcinoma (PDAC) is a highly lethal malignancy. Next-generation sequencing (NGS) enables molecular characterization and may identify clinically actionable alterations; however, real-world multicenter data linking genomic subgroups to survival outcomes remain limited. We aimed to characterize the molecular landscape of NGS-tested PDAC in a Turkish multicenter cohort and evaluate the association of key molecular alterations, including *KRAS* status and *KRAS* variant subgroups, with survival outcomes. **Methods:** We conducted a multicenter retrospective cohort study including patients with pathologically diagnosed PDAC between 2017 and 2025 who underwent tumor-based NGS in routine clinical practice. Overall survival (OS) was calculated from the date of metastasis, defined as the date of diagnosis for de novo metastatic disease and the date of first documented distant recurrence for recurrent cases. Progression-free survival (PFS) was calculated from the initiation of first-line systemic therapy for metastatic disease to progression or death. Survival was estimated using the Kaplan–Meier method and compared using the log-rank test. Multivariable Cox proportional hazards models were constructed for OS and PFS using clinically relevant covariates selected a priori. **Results:** A total of 98 patients underwent molecular profiling, and survival analyses were performed in 92 patients with available OS/PFS data. *KRAS* mutations were detected in 83.7% (82/98) of patients, with predominant variants G12D (47.6%), G12V (30.5%), and G12R (12.2%). *TP53* mutations were present in 59.2% (58/98) of tumors, and all tumors were microsatellite stable. Tumor mutational burden data were available for 72 patients; the median TMB was 3.83 mutations/Mb, and 15.3% of evaluable tumors had a TMB ≥ 10 mutations/Mb. Excluding *KRAS*, clinically actionable alterations were identified in 4.1% of patients, whereas an additional 32.7% harbored potentially actionable or investigational alterations. Median OS was 14.0 months (95% CI, 11.7–16.3), and median PFS was 6.0 months (95% CI, 4.3–7.7). In unadjusted analyses, OS and PFS did not differ significantly according to *KRAS* mutation status (OS, *p* = 0.967; PFS, *p* = 0.652), TP53 mutation status (OS, *p* = 0.404; PFS, *p* = 0.510), or *KRAS* variant subgroup (OS, *p* = 0.332; PFS, *p* = 0.194). In multivariable Cox analyses, *KRAS* mutation status was not independently associated with OS (aHR 1.13, 95% CI 0.56–2.28; *p* = 0.727) or PFS (aHR 1.09, 95% CI 0.59–2.01; *p* = 0.780), whereas ECOG performance status remained the strongest adverse clinical factor. **Conclusions:** In this multicenter real-world PDAC cohort, the molecular landscape was dominated by *KRAS* and *TP53* alterations, whereas clinically actionable non-*KRAS* alterations were identified in only a minority of patients. After adjustment for major clinical covariates, *KRAS* mutation status was not independently associated with OS or PFS. Molecular profiling may still be useful for identifying uncommon potentially targetable alterations; however, larger clinically annotated multicenter studies are needed to better define its prognostic and treatment-directing value in routine practice.

## Simple Summary

Pancreatic ductal adenocarcinoma is one of the deadliest cancers, and most patients with metastatic disease have limited treatment options. Molecular testing may identify genetic alterations that could help guide targeted treatment in a small subset of patients, but its real-world clinical value remains uncertain. In this multicenter study from Turkey, we analyzed tumor-based next-generation sequencing results from 98 patients with metastatic pancreatic cancer. Most tumors carried KRAS and TP53 alterations, while clearly actionable non-KRAS alterations were uncommon. Survival did not differ significantly according to KRAS mutation status or KRAS variant subgroup, and in adjusted analyses, clinical factors such as performance status were more strongly associated with outcome than the genomic variables examined. These findings support the role of molecular profiling for identifying rare targetable alterations, while also showing the need for larger clinically annotated real-world datasets.

## 1. Introduction

Despite advances in systemic therapy and supportive care, pancreatic ductal adenocarcinoma (PDAC) remains a major global health challenge, characterized by disproportionate mortality relative to its incidence and persistently poor long-term outcomes. Contemporary global estimates (GLOBOCAN 2022) underscore the continuing burden of pancreatic cancer worldwide, reinforcing the urgent need for strategies to improve both survival and therapeutic precision [1]. Given the lethality of PDAC and the narrow window for curative-intent treatment, improving earlier diagnosis remains a major unmet need alongside advances in systemic and biomarker-driven therapy.

In metastatic PDAC, modern combination chemotherapy regimens, such as FOLFIRINOX and gemcitabine plus nab-paclitaxel, have established survival benefits over gemcitabine alone; however, the majority of patients ultimately experience progression, and durable disease control remains uncommon [2,3]. This therapeutic ceiling has catalyzed a shift toward biomarker-driven approaches aimed at identifying clinically meaningful subgroups that may benefit from targeted agents or immunotherapy, as reflected in international clinical practice guidelines [4,5].

Large-scale genomic studies have defined a recurrent molecular backbone in PDAC, dominated by alterations in *KRAS, TP53, CDKN2A,* and *SMAD4*, alongside less frequent but potentially actionable aberrations in DNA repair pathways and other oncogenic drivers [6,7,8]. Early pathway-level analyses further demonstrated that pancreatic cancers harbor numerous genetic alterations converging on a core set of signaling pathways and cellular processes, supporting the concept that molecular heterogeneity may translate into clinically relevant phenotypes [9]. In addition, evolutionary studies of PDAC suggest substantial intratumoral heterogeneity and clonal dynamics that may influence patterns of dissemination and treatment resistance [10].

Among the actionable biomarkers, defects in homologous recombination repair (HRR) and broader DNA damage response (DDR) pathways represent one of the most clinically consequential axes in PDAC [11,12]. The POLO trial provided Level-1 evidence that maintenance PARP inhibition (olaparib) can improve progression-free survival in patients with germline BRCA-mutated metastatic PDAC who do not progress on first-line platinum-based chemotherapy [13,14,15]. In addition, precision oncology registry data suggest that molecular profiling followed by matched therapy may be associated with improved survival in selected patients, supporting the clinical value of identifying actionable alterations in routine practice [16,17,18].

Although PDAC is generally considered an immunologically “cold” tumor, a small subset characterized by mismatch repair deficiency/high microsatellite instability (dMMR/MSI-H) can derive meaningful benefit from PD-1 blockade. Foundational clinical evidence established that mismatch repair deficiency predicts benefit from pembrolizumab across tumor types, providing a rationale for routine MSI/dMMR assessment when feasible [19,20,21]. Beyond immunotherapy, rare but high-impact oncogenic fusions, such as *NTRK*, can enable tissue-agnostic targeted therapy, with larotrectinib demonstrating robust and durable activity in *TRK* fusion-positive cancers irrespective of tumor origin [22]. Collectively, these data underpin guideline recommendations advocating comprehensive molecular profiling (including DDR/HRR genes, MSI/dMMR, and selected fusions) in advanced PDAC, where results may influence treatment selection or trial enrollment [4,5].

Real-world next-generation sequencing (NGS) cohorts have reported variable, yet clinically meaningful, frequencies of potentially actionable alterations, which are influenced by patient selection, panel content, specimen adequacy, and access to matched therapies. This point is further supported by published real-world NGS cohorts; for example, a targeted NGS study in a Korean cohort reported that approximately one-third of patients harbored potentially actionable alterations, with highly actionable findings enriched in DNA damage repair (DDR) genes [18]. Furthermore, a clinically selected European tissue-based NGS cohort demonstrated the feasibility of integrating molecular profiling into therapeutic decision-making, including the identification of targetable lesions, such as *BRCA1/2* alterations, dMMR, and *NRG1* fusions [23]. In parallel, emerging multi-cohort analyses have highlighted that *KRAS* is not a binary marker; rather, *KRAS* allelic subtypes and co-mutation patterns may stratify prognosis and biology, suggesting additional value from granular genomic annotation beyond “KRAS-mutant” status alone [24].

Despite these advances, important gaps remain in translating genomic profiling into consistent survival gains at the population level, particularly across different healthcare systems and real-world settings. Regional multicenter datasets are therefore critical to (i) define the distribution of actionable and prognostic alterations, (ii) describe how often profiling leads to matched therapy, and (iii) quantify associations between genomic features and survival outcomes under routine care conditions. In this context, we conducted a multicenter Turkish real-world study of patients with PDAC diagnosed between 2017 and 2025 who underwent next-generation sequencing, aiming to characterize the molecular genomic landscape and evaluate its association with survival outcomes in routine clinical practice.

## 2. Materials and Methods

### 2.1. Study Design and Setting

We conducted a multicenter retrospective cohort study across participating oncology centers in Turkey. The study period spanned January 2017 to December 2025 and included patients managed in routine clinical practice who underwent tumor-based next-generation sequencing (NGS) for pancreatic cancer. NGS testing was performed as part of routine clinical care rather than under a standardized prospective protocol.

### 2.2. Participants

Adult patients (≥18 years) with histopathologically confirmed pancreatic ductal adenocarcinoma (PDAC) were eligible if they had metastatic disease at diagnosis or developed metastatic disease during follow-up and had an available tumor NGS report. Patients with non-PDAC pancreatic malignancies or non-pancreatic primaries were excluded. All consecutive eligible NGS-tested patients meeting these criteria during the study period were included. Because tumor NGS was not universally performed in all patients with metastatic PDAC during routine practice and was ordered according to real-world clinical practice rather than a uniform prospective protocol, the final cohort represents a clinically selected NGS-tested metastatic PDAC population rather than an unselected metastatic PDAC population.

### 2.3. Data Sources and Variables

Clinical, pathological, and treatment-related data were retrospectively extracted from institutional electronic medical records using a standardized data abstraction form. The collected variables comprised demographic characteristics, ECOG performance status, smoking/alcohol history, tumor stage at diagnosis, de novo metastatic presentation, primary tumor location, metastatic sites and number of metastatic sites, systemic treatment patterns (including first-line palliative chemotherapy regimen and treatment line), and follow-up status. Molecular variables were abstracted from NGS laboratory reports and included *KRAS* mutation status (mutant vs. wild-type), *KRAS* variant subgroup classification (G12D, G12V, G12R, Q61H/others), and selected recurrent co-alterations commonly reported in PDAC (e.g., *TP53, CDKN2A, SMAD4*, and DNA damage repair genes such as *BRCA2* and *ATM*, where available). Microsatellite status (MSS vs. MSI-H) and tumor mutational burden (TMB, mutations per megabase) were recorded when reported in the NGS output. TMB was summarized as a continuous variable and additionally categorized using a prespecified threshold of ≥10 mutations/Mb to define TMB-high tumors. Detailed matched targeted-therapy delivery was not systematically available across all participating centers.

#### Clinical Actionability Classification

Clinical actionability was assigned at the patient level according to the highest-ranking non-*KRAS* alteration identified using a modified evidence framework informed by the ESMO Scale for Clinical Actionability of Molecular Targets (ESCAT) and current tumor-agnostic approvals. Non-*KRAS* alterations were categorized as clinically actionable, potentially actionable/investigational, or non-actionable/prognostic. Alteration type was incorporated into the classification whenever relevant (e.g., mutation, amplification, fusion, exon 14 skipping). Patients harboring more than one non-*KRAS* alteration were assigned to the highest actionability category. *MLH1* alteration alone was not considered clinically actionable in the absence of a confirmed dMMR/MSI-H status, and the *NTRK2* point mutation was not considered equivalent to an *NTRK* fusion.

### 2.4. Tumor Tissue Acquisition and NGS Testing

Tumor tissue for NGS was obtained from the primary pancreatic tumor or metastatic sites, depending on clinical availability and standard diagnostic workflow at each center. Tumor sequencing was performed using locally available targeted panels rather than a single centralized assay. Because this study reflects real-world practice, testing platforms and reporting elements (including MSI/TMB) were not uniform across all cases; therefore, biomarker-specific denominators are reported as the number of patients with available results.

### 2.5. Outcomes

The primary endpoint was overall survival (OS), defined as the time from the onset of metastatic disease to death from any cause. For patients with de novo metastatic PDAC, the metastatic index date was the date of diagnosis; for patients initially diagnosed with non-metastatic disease who subsequently developed distant recurrence, the metastatic index date was the date of the first documented metastatic relapse. Patients who were alive at the last follow-up were censored on the date of last contact.

The secondary endpoint was progression-free survival (PFS), defined as the time from the initiation of first-line systemic therapy for metastatic disease to radiologic or clinical progression or death, whichever occurred first. For patients who did not receive systemic therapy for metastatic disease, PFS was calculated from the date of metastatic index to progression/death when applicable; otherwise, patients were censored at the last disease assessment.

This approach was chosen to align the time origin to the metastatic disease phase across the cohort; however, potential clinical heterogeneity between de novo and recurrent metastatic cases was considered in adjusted analyses.

### 2.6. Statistical Analysis

Baseline characteristics were summarized using descriptive statistics; categorical variables were presented as counts and percentages, while continuous variables were summarized as mean (standard deviation) or median (interquartile range), depending on the distribution. OS and PFS were estimated using the Kaplan–Meier method. Survival curves were compared using the log-rank test according to *KRAS* mutation status and *KRAS* variant subgroups (G12D, G12V, G12R, Q61H/others), and additional exploratory analyses were performed for *TP53* status where relevant. To evaluate the independent association of selected molecular variables with survival outcomes, multivariable Cox proportional hazards models were constructed for OS and PFS using clinically relevant covariates selected a priori, including age (continuous), ECOG performance status (0–1 vs. ≥2), disease presentation (de novo metastatic vs. recurrent metastatic), primary tumor location (head vs. non-head), metastatic burden (<2 vs. ≥2 metastatic sites), and first-line treatment intensity (FOLFIRINOX vs. non-FOLFIRINOX). Separate adjusted models were fitted for *KRAS* mutation status and *TP53* mutation status. Because of limited subgroup size, *KRAS* allele-specific subgroup analyses were interpreted as exploratory, and nominal *p*-values were interpreted descriptively. The proportional hazards assumption was assessed and no major violation was observed. All statistical tests were two-sided, and *p*-values < 0.05 were considered statistically significant. Analyses were performed using IBM SPSS Statistics for Windows, version 23 (IBM Corp., Armonk, NY, USA).

### 2.7. Ethics Approval

This study was approved by the Memorial Antalya Hospital Clinical Research Ethics Committee (approval no.: 847/2025, date: 20 October 2025). Given its retrospective, non-interventional design and de-identified data, the requirement for informed consent was waived. This study complied with the principles of the Declaration of Helsinki and local regulations.

## 3. Results

### 3.1. Patient Characteristics

We included 98 patients with histologically confirmed pancreatic ductal adenocarcinoma (PDAC) who underwent tumor-based next-generation sequencing (NGS) (Table 1). The median age at diagnosis was 61 years (interquartile range [IQR], 55–69 years), and 54.1% were men (Table 1). At initial diagnosis, 64.3% of patients had stage IV disease and 64.3% presented with de novo metastasis (Table 1). The pancreatic head was the most common primary tumor location (46.9%), followed by the body (21.4%) and tail (16.3%) (Table 1).

With respect to the metastatic burden, 44.9% of patients had one metastatic site, 27.6% had two sites, and 20.4% had ≥3 sites (Table 1). The liver was the most frequent metastatic site (65.3%), followed by the peritoneum (31.6%), lung (18.4%), bone (12.2%), and non-regional nodal metastases (35.7%) (Table 1). First-line palliative chemotherapy predominantly comprised FOLFIRINOX (61.2%) and gemcitabine-based regimens (26.5%) (Table 1).

### 3.2. Molecular Genomic Landscape and Actionability

**Figure 1:**
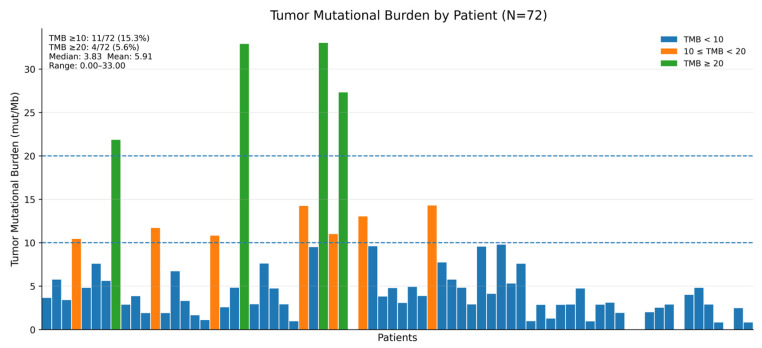
Distribution of tumor mutational burden in evaluable patients. Bar plot showing the distribution of tumor mutational burden (TMB) in evaluable patients with pancreatic ductal adenocarcinoma (N = 72). The median TMB was 3.83 mutations/Mb (mean, 5.91; range, 0.00–33.00). Eleven patients (15.3%) had TMB ≥ 10 mutations/Mb, and four patients (5.6%) had TMB ≥ 20 mutations/Mb. The dashed horizontal lines indicate the TMB cut-off values of 10 mut/Mb and 20 mut/Mb.

*KRAS* mutations were detected in 82 of 98 (83.7%) patients, whereas 16 of 98 (16.3%) were *KRAS* wild-type (Table 2). Among the *KRAS*-mutated tumors, the most common *KRAS* variants were G12D (47.6%), G12V (30.5%), G12R (12.2%), and Q61H and other variants (9.8%) (Table 2). *TP53* mutations were present in 58 of 98 (59.2%) patients, followed by *CDKN2A* (11.2%) and *SMAD4* (5.1%) (Table 2). All tumors were microsatellite stable (MSS) (Table 2). Tumor mutational burden (TMB) data were available for 72 patients because TMB reporting was not uniform across the local NGS panels and reports used in routine practice. The median TMB was 3.83 mutations/Mb (mean, 5.91; range, 0.00–33.00). TMB was ≥10 mutations/Mb in 11 of 72 patients (15.3%), whereas four patients (5.6%) had TMB ≥20 mutations/Mb (Figure 1). The full catalog of alterations, including low-frequency mutations and copy-number alterations, is provided in Table S1.

**Figure 2:**
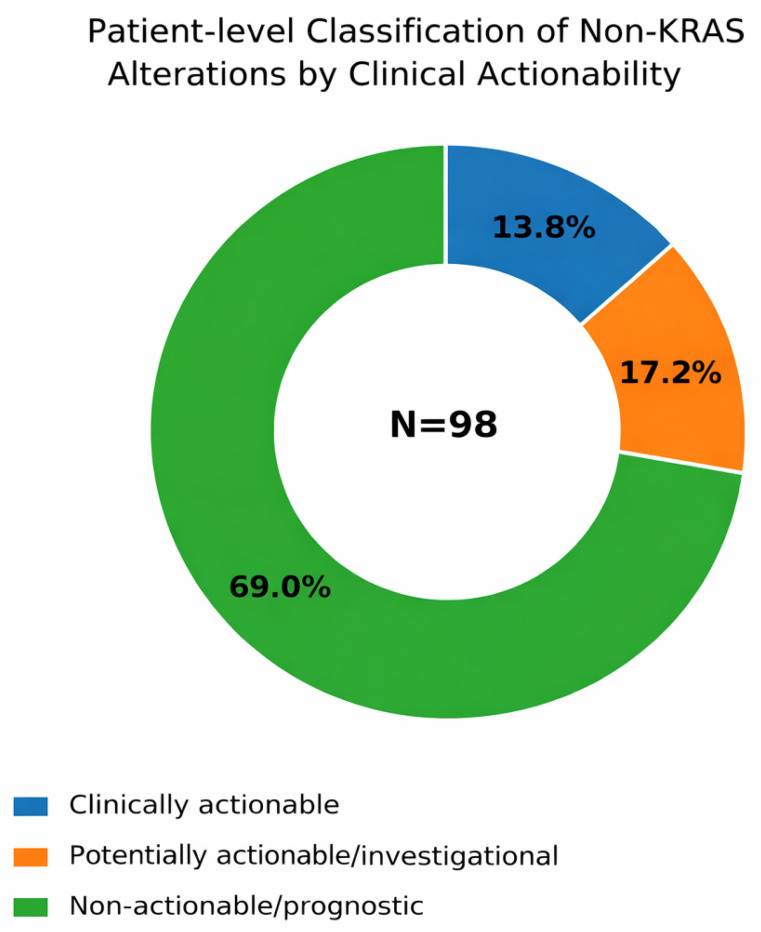
Patient-level classification of non-*KRAS* alterations by clinical actionability. Donut chart showing the distribution of non-*KRAS* alterations according to the highest level of clinical actionability assigned at the patient level in the overall cohort (N = 98). Alterations were categorized as clinically actionable, potentially actionable/investigational, or non-actionable/prognostic.

Excluding *KRAS*, clinically actionable alterations were identified in 4.1% of patients (n = 4), consisting of *BRCA2* mutations and *ROS1* fusion. An additional 32.7% of patients (n = 32) harbored potentially actionable/investigational alterations with possible relevance for off-label treatment consideration or clinical trial enrollment, including alterations affecting DDR, cell cycle, HER2/RTK, PI3K/AKT/mTOR, and MAPK-related pathways. The remaining 63.3% of patients (n = 62) were classified as non-actionable/prognostic only according to the highest-ranked non-*KRAS* alteration detected (Table S2; Figure 2).

**Figure 3:**
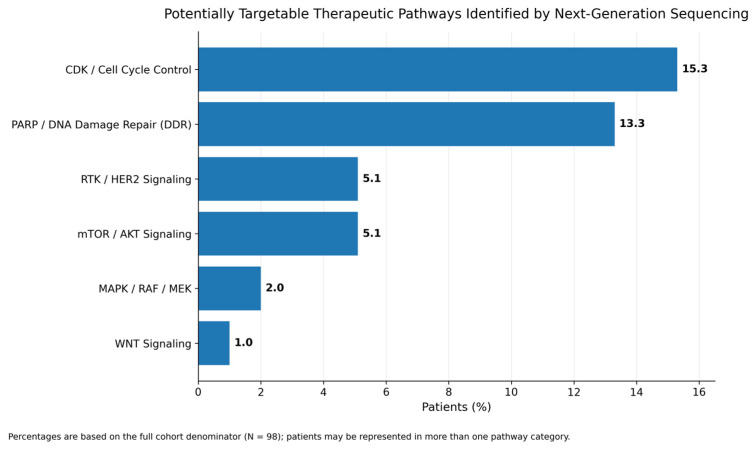
Potentially targetable therapeutic pathways identified by NGS. Bar plot showing the frequency of potentially targetable therapeutic pathways identified by next-generation sequencing in the overall cohort. Percentages are based on the full cohort denominator (N = 98), and patients with more than one alteration may be represented in multiple pathway categories.

Pathway-based grouping demonstrated that potentially actionable/investigational alterations most commonly involved CDK/cell cycle control and DDR/PARP-related pathways, followed by RTK/HER2, mTOR/AKT, MAPK/RAF/MEK, and WNT signaling (Table S3; Figure 3).

### 3.3. Overall Survival

**Figure 4:**
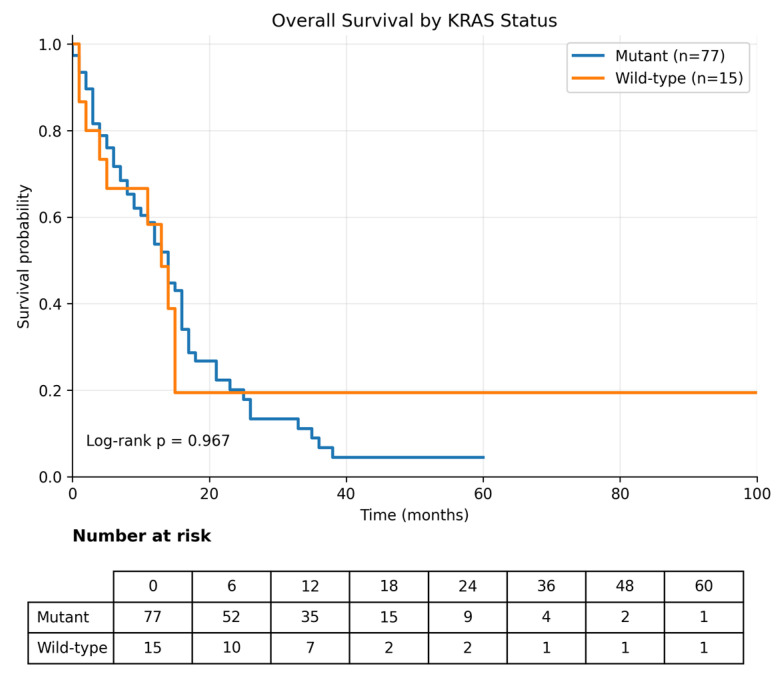
Overall survival according to *KRAS* mutation status. Kaplan–Meier analysis of overall survival stratified by *KRAS* mutation status (mutant vs. wild-type). No significant difference in overall survival was observed between the groups (log-rank *p* = 0.967).

**Figure 5:**
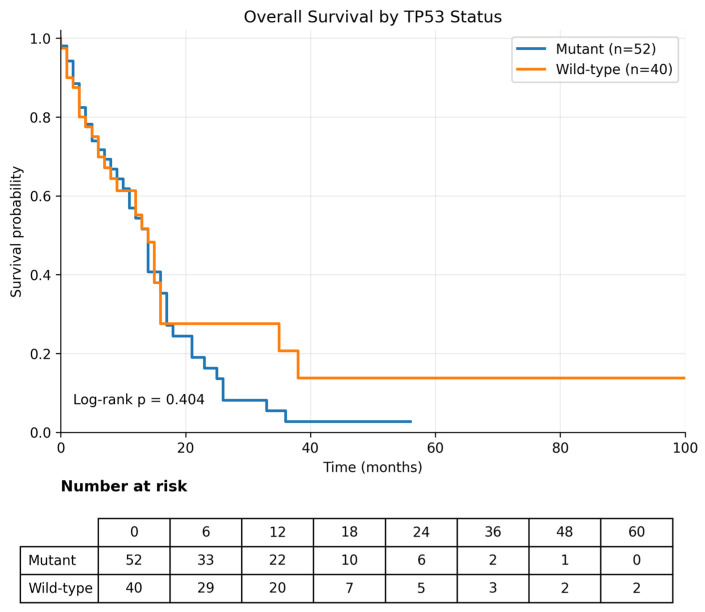
Overall survival according to *TP53* mutation status. Kaplan–Meier analysis of overall survival stratified by *TP53* mutation status (mutant vs. wild-type). No significant difference in overall survival was observed between the groups (log-rank *p* = 0.404).

Overall survival (OS) was calculated from the metastatic index date (diagnosis date for de novo metastatic disease and date of first documented distant recurrence for recurrent metastatic disease). Analyses were performed in 92 patients. During follow-up, 67 deaths were recorded, and the median OS for the overall cohort was 14.0 months (95% CI, 11.7–16.3) (Table 3). When stratified by *KRAS* mutation status, the median OS was 13.0 months (95% CI, 8.7–17.3) in patients with *KRAS* wild-type tumors and 14.0 months (95% CI, 11.4–16.6) in patients with *KRAS*-mutant tumors, with no statistically significant difference between the groups (log-rank *p* = 0.967) (Table 3; Figure 4). Likewise, *TP53* mutation status was not significantly associated with OS; the median OS was 14.0 months (95% CI, 11.1–16.9) in *TP53* wild-type tumors and 14.0 months (95% CI, 11.2–16.8) in *TP53*-mutant tumors (log-rank *p* = 0.404) (Table 3; Figure 5).

### 3.4. Progression-Free Survival

**Figure 6:**
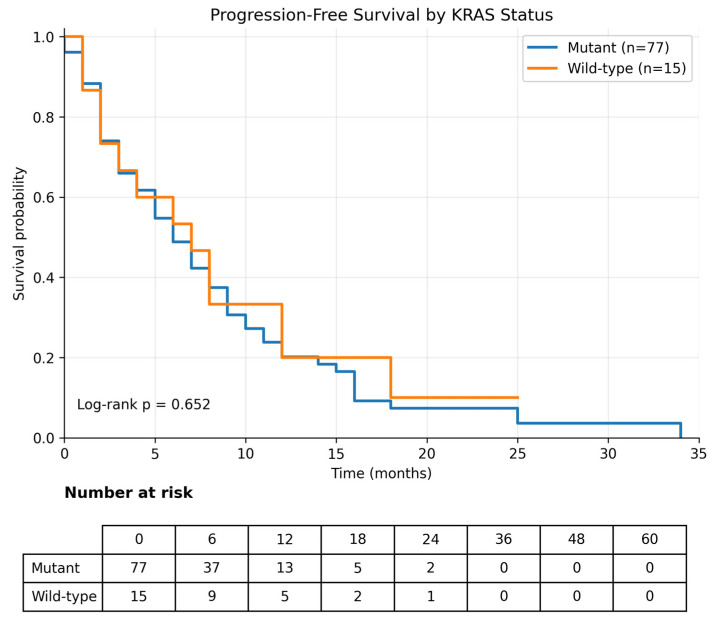
Progression-free survival according to *KRAS* mutation status. Kaplan–Meier analysis of progression-free survival stratified by *KRAS* mutation status (mutant vs. wild-type). No significant difference in progression-free survival was observed between the groups (log-rank *p* = 0.652).

**Figure 7:**
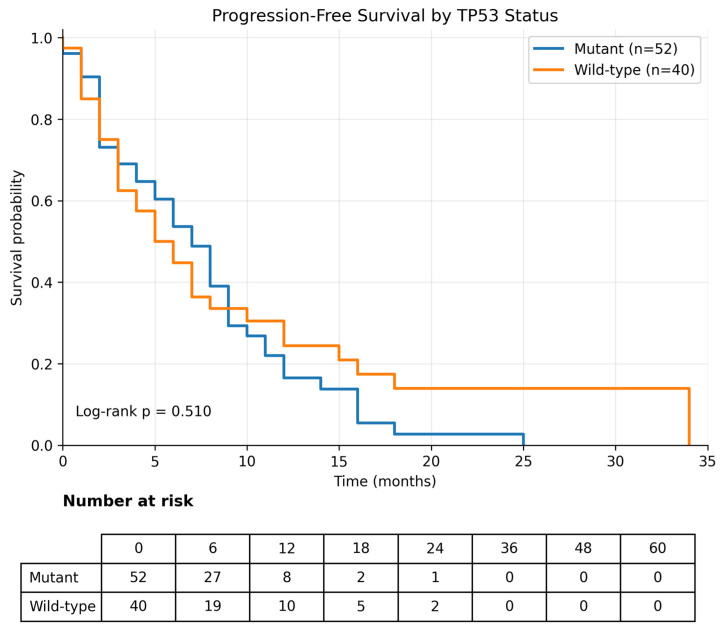
Progression-free survival according to *TP53* mutation status. Kaplan–Meier analysis of progression-free survival stratified by *TP53* mutation status (mutant vs. wild-type). No significant difference in progression-free survival was observed between the groups (log-rank *p* = 0.510).

Progression-free survival (PFS) was calculated from the initiation of first-line systemic therapy for metastatic disease to progression or death. Analyses were performed in 92 patients. A total of 77 progression events were observed, and the median PFS for the overall cohort was 6.0 months (95% CI, 4.3–7.7) (Table 4). No significant difference in PFS was observed according to *KRAS* mutation status; the median PFS was 7.0 months (95% CI, 3.2–10.8) in *KRAS* wild-type tumors and 6.0 months (95% CI, 4.2–7.8) in *KRAS*-mutant tumors (log-rank *p* = 0.652) (Table 4; Figure 6). Similarly, *TP53* mutation status was not significantly associated with PFS; the median PFS was 5.0 months (95% CI, 2.6–7.4) in *TP53* wild-type tumors and 7.0 months (95% CI, 5.0–9.0) in *TP53*-mutant tumors (log-rank *p* = 0.510) (Table 4; Figure 7).

### 3.5. Survival According to KRAS Variant Subgroups

**Figure 8:**
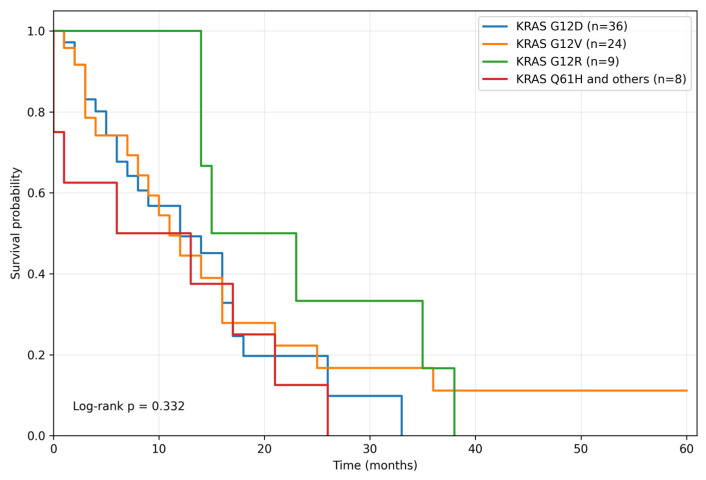
Overall survival according to *KRAS* variant subgroups. Kaplan–Meier analysis of overall survival among *KRAS*-mutated patients stratified by *KRAS* variant subgroup. No significant difference in overall survival was observed across subgroups (log-rank *p* = 0.332).

**Figure 9:**
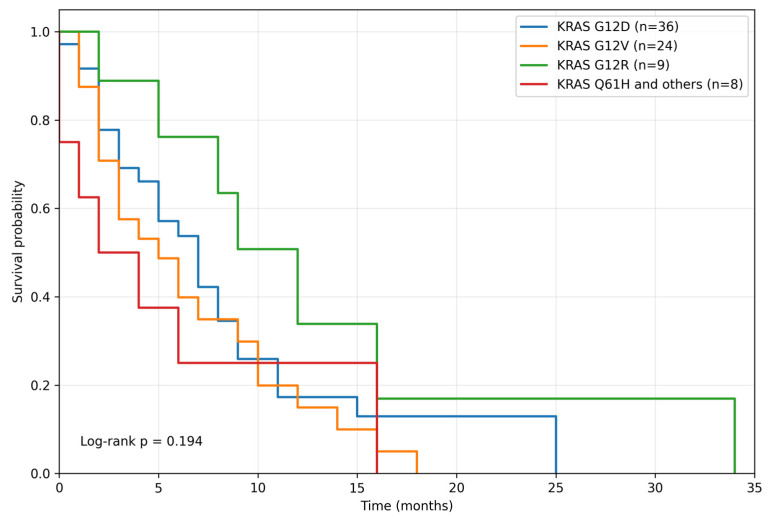
Progression-free survival according to *KRAS* variant subgroups. Kaplan–Meier analysis of progression-free survival among *KRAS*-mutated patients stratified by *KRAS* variant subgroup. No significant difference in progression-free survival was observed across subgroups (log-rank *p* = 0.194).

Survival outcomes were evaluated among patients with *KRAS* mutations and available survival data (n = 77), according to *KRAS* variant subgroups: G12D (n = 36), G12V (n = 24), G12R (n = 9), and Q61H and other variants (n = 8). Overall survival (OS) did not differ significantly across *KRAS* variant subgroups (log-rank *p* = 0.332) (Figure 8). The median OS was 12.0 months for *KRAS* G12D, 11.0 months for *KRAS* G12V, 15.0 months for *KRAS* G12R, and 6.0 months for *KRAS* Q61H/others. Similarly, progression-free survival (PFS) did not differ significantly across *KRAS* variant subgroups (log-rank *p* = 0.194) (Figure 9). The median PFS was 7.0 months for *KRAS* G12D, 5.0 months for *KRAS* G12V, 12.0 months for *KRAS* G12R, and 2.0 months for *KRAS* Q61H/others.

### 3.6. Multivariable Survival Analyses

Multivariable Cox proportional hazards analyses were performed for both OS and PFS in the 92 patients with available survival data. In the adjusted model including age, ECOG performance status, disease presentation, primary tumor location, metastatic burden, and first-line treatment intensity, *KRAS* mutation status was not independently associated with OS (aHR 1.13, 95% CI 0.56–2.28, *p* = 0.727) or PFS (aHR 1.09, 95% CI 0.59–2.01, *p* = 0.780). Similar findings were observed for *TP53* mutation status, which was not independently associated with OS (aHR 1.32, 95% CI 0.78–2.23, *p* = 0.309) or PFS (aHR 1.13, 95% CI 0.70–1.82, *p* = 0.616). Among the clinical covariates, ECOG ≥ 2 remained independently associated with worse OS (aHR 4.39, 95% CI 2.04–9.42, *p* < 0.001) and PFS (aHR 3.26, 95% CI 1.55–6.87, *p* = 0.002). *KRAS* allele-specific subgroup analyses remained exploratory because of limited subgroup size (Table 5 and Table 6; Supplementary Tables S4 and S5).

## 4. Discussion

In this multicenter real-world cohort of patients with pancreatic ductal adenocarcinoma (PDAC) who underwent next-generation sequencing (NGS), we characterized the molecular landscape and evaluated the prognostic impact of selected molecular alterations. The principal findings are as follows: (i) the molecular architecture of our cohort recapitulated the canonical PDAC genomic backbone, dominated by *KRAS* and *TP53* alterations; (ii) a minority of patients harbored potentially actionable genomic alterations, particularly within DNA damage response (DDR) pathways; and (iii) after adjustment for major available clinical covariates, *KRAS* mutation status was not independently associated with overall survival (OS) or progression-free survival (PFS), while ECOG performance status remained the strongest adverse clinical factor.

The high prevalence of *KRAS* mutations observed in our study is consistent with large-scale genomic analyses, including The Cancer Genome Atlas (TCGA) and whole-genome sequencing efforts, which established *KRAS* as a central driver in approximately 80–90% of PDAC cases [6,7,8]. Similarly frequent co-occurring *TP53* alterations reinforce the conserved genomic framework described in high-impact molecular profiling studies [6,7]. Our data confirm, within a Turkish multicenter real-world population, the reproducibility of the molecular backbone originally defined in genomic consortia.

A central objective of this study was to investigate whether *KRAS* allelic subtypes confer differential survival outcomes. Although mechanistic and pathway-level studies suggest that specific *KRAS* variants may activate downstream signaling networks differently, recent clinical and multi-cohort analyses have further indicated survival heterogeneity according to *KRAS* allele and co-mutation patterns [24,25]; however, our adjusted analyses did not demonstrate an independent association between *KRAS* mutation status and OS or PFS in this clinically selected real-world cohort. At the same time, the limited size of the *KRAS* wild-type and allele-specific subgroups constrains statistical power and leaves open the possibility of false-negative subgroup findings.

Several factors may account for the lack of significant survival discrimination. First, metastatic PDAC outcomes are strongly influenced by performance status, metastatic burden, and treatment intensity, potentially attenuating allele-specific biological effects in routine practice. Second, subgroup sample sizes—particularly for less common variants—limit the statistical power to detect modest effect sizes. Third, real-world cohorts reflect therapeutic heterogeneity, variable timing of molecular testing, and residual/unmeasured confounding, which may dilute signals observed in highly curated datasets. Collectively, these considerations suggest that genomic variables should be interpreted in clinical context rather than as standalone prognostic markers. In the adjusted models, baseline clinical condition, particularly ECOG performance status, appeared to exert a stronger influence on outcome than the genomic variables examined here.

From a therapeutic perspective, our study identified a clinically meaningful subset of patients harboring potentially actionable alterations, most notably within the DDR pathways. This observation aligns with prior reports demonstrating enrichment of DDR gene alterations in advanced PDAC cohorts [6]. Importantly, actionability in our study was defined using a modified ESCAT-informed framework adapted to PDAC and tumor-agnostic approvals. Therefore, only alterations with established therapeutic relevance were classified as clinically actionable, whereas alterations such as *ATM* and other DDR-related events were considered potentially actionable/investigational rather than routinely targetable in current PDAC practice. The clinical relevance of such findings is underscored by the POLO trial, which demonstrated improved progression-free survival with maintenance olaparib in germline *BRCA*-mutated metastatic PDAC following platinum-based therapy [13]. Furthermore, registry-based precision oncology data suggest that molecularly matched therapies may be associated with improved survival in selected patients [26]. Although our study was not designed to evaluate matched therapy outcomes specifically, the detection of DDR alterations reinforces the rationale for comprehensive molecular profiling in metastatic PDAC. Accordingly, these findings should be interpreted primarily as a rationale for referral to molecular tumor boards, treatment individualization, and clinical trial consideration rather than as direct evidence of routine matched therapy benefit. Importantly, this study does not demonstrate that molecular profiling directly influenced treatment selection or improved survival outcomes in this cohort.

TMB levels were evaluable in 72 patients and were generally low overall, with a median value of 3.83 mutations/Mb. Although a minority of tumors had TMB ≥ 10 mutations/Mb, all cases were microsatellite stable, and the clinical relevance of TMB in PDAC remains uncertain outside of mismatch repair-deficient settings [27,28,29]. Foundational evidence has demonstrated that PD-1 blockade yields meaningful benefit in tumors with mismatch-repair deficiency across cancer types [19]; however, the rarity of MSI-H PDAC limits the proportion of patients eligible for immune checkpoint inhibition under current biomarker paradigms.

Importantly, our study provides region-specific real-world genomic data from Turkey, complementing a literature largely derived from Western cohorts. The inclusion of both de novo metastatic and recurrent metastatic PDAC enhances the clinical relevance of our findings to routine practice. Furthermore, by anchoring survival analyses to the metastatic disease phase, we aimed to reduce potential lead-time bias and align the survival time origin across the cohort, although residual clinical heterogeneity between de novo and recurrent metastatic cases remains possible.

This study has several limitations. First, the retrospective multicenter design introduces potential selection bias because only patients who underwent tumor NGS in routine practice were included, and NGS was not ordered according to a uniform prospective protocol. Second, the cohort size was modest for genomic subgroup analyses, particularly for *KRAS* wild-type and allele-specific subgroups. Third, tumor sequencing was performed using non-uniform local panels, which may have influenced biomarker availability and detection rates, particularly for TMB. Fourth, although the revised analyses adjusted for major available clinical covariates, residual confounding related to treatment selection and other unmeasured variables remains possible. Finally, the study did not systematically capture whether clinically actionable alterations led to matched targeted therapy, and therefore clinical actionability should not be interpreted as equivalent to confirmed treatment delivery.

## 5. Conclusions

In conclusion, in this multicenter real-world metastatic PDAC cohort, the molecular landscape was dominated by *KRAS* and *TP53* alterations, whereas clinically actionable non-*KRAS* alterations were identified in only a minority of patients. After adjustment for major clinical covariates, *KRAS* mutation status was not independently associated with OS or PFS, and similar findings were observed for *TP53* mutation status. Molecular profiling may be useful for identifying uncommon potentially targetable alterations; however, its direct impact on clinical decision-making and patient outcomes remains uncertain in this real-world setting.

## Figures and Tables

**Table 1:** Baseline demographic, clinicopathological, and treatment characteristics of the study cohort (*N* = 98).

Variable	Category	Total
Age, years	Median (IQR)	61 (55–69)
	<65	58 (59.2)
	≥65	40 (40.8)
Sex, n (%)	Female	45 (45.9)
	Male	53 (54.1)
ECOG performance status, n (%)	0	54 (55.1)
	1	34 (34.7)
	2	10 (10.2)
Stage at diagnosis, n (%)	II	19 (19.4)
	III	16 (16.3)
	IV	63 (64.3)
De novo metastatic disease, n (%)	Yes	63 (64.3)
Primary tumor location, n (%)	Head	46 (46.9)
	Body	21 (21.4)
	Tail	16 (16.3)
	Overlapping/unknown	15 (15.3)
Histology, n (%)	Adenocarcinoma	96 (98.0)
	Mucinous carcinoma	1 (1.0)
	Unknown	1 (1.0)
Number of metastatic sites, n (%)	0 †	7 (7.1)
	1	44 (44.9)
	2	27 (27.6)
	≥3	20 (20.4)
Metastatic sites, n (%) *	Liver	64 (65.3)
	Peritoneum	31 (31.6)
	Lung	18 (18.4)
	Bone	12 (12.2)
	Non-regional lymph nodes	35 (35.7)
First-line palliative chemotherapy, n (%)	FOLFIRINOX	60 (61.2)
	Gemcitabine-based combination	26 (26.5)
	Gemcitabine monotherapy	3 (3.1)
	Other	2 (2.0)
	No first-line palliative chemotherapy	7 (7.1)

Data are presented as n (%) unless otherwise indicated. Abbreviations: ECOG, Eastern Cooperative Oncology Group; IQR, interquartile range. * Metastatic site frequencies are not mutually exclusive; therefore, the percentages may sum to more than 100%. † Patients categorized as having zero metastatic sites represent recurrent metastatic cases in whom site-count data at the metastatic index date were unavailable or separately coded.

**Table 2:** Key genomic alterations identified by next-generation sequencing (NGS) (*N* = 98).

Genes/Biomarker	Category	n (%)
***KRAS***		
	Mutation	82 (83.7)
	Wild-type	16 (16.3)
***KRAS*** ** Variant**		
	G12D	39 (47.6)
	G12V	25 (30.5)
	G12R	10 (12.2)
	Q61H and others	8 (9.8)
***TP53***	Mutation	58 (59.2)
***CDKN2A***	Mutation	11 (11.2)
***SMAD4***	Mutation	5 (5.1)
***ATM***	Mutation	4 (4.1)
***BRCA2***	Mutation	3 (3.1)
***ARID1A***	Mutation	3 (3.1)
***GNAS8***	Mutation	3 (3.1)
***SF3B1***	Mutation	3 (3.1)
***STK11***	Mutation	2 (2.0)
***FANCA***	Mutation	2 (2.0)
***FBXW7***	Mutation	2 (2.0)
***MCL1***	Mutation	2 (2.0)
***ERBB2***	Amplification	1 (1.0)
***ERBB2***	Mutation	1 (1.0)
***MET*** ** exon 14 skipping**	Mutation	1 (1.0)
***MLH1***	Mutation	1 (1.0)
***NTRK2***	Mutation	1 (1.0)
***PIK3CA***	Mutation	1 (1.0)
***PMS1***	Mutation	1 (1.0)
***RNF43***	Mutation	1 (1.0)
***ROS1***	Fusion	1 (1.0)
***TGFBR2***	Mutation	1 (1.0)
**MSI status**	MSS	98 (100.0)

Abbreviations: MSI, microsatellite instability; MSS, microsatellite stable; NGS, next-generation sequencing.

**Table 3:** Overall survival (OS) according to *KRAS* and *TP53* status.

Group		N	Events	Median OS, Months (95% CI)	*p*
**Overall**		92	67	14.0 (11.7–16.3)	
***KRAS***					0.967
	Wild-type	15	10	13.0 (8.7–17.3)	
	Mutant	77	57	14.0 (11.4–16.6)	
***TP53***					0.404
	Wild-type	40	27	14.0 (11.1–16.9)	
	Mutant	52	40	14.0 (11.2–16.8)	

Abbreviations: CI, confidence interval; OS, overall survival.

**Table 4:** Progression-free survival (PFS) according to *KRAS* and *TP53* status.

Group		N	Events	Median PFS, Months (95% CI)	*p*
**Overall**		92	77	6.0 (4.3–7.7)	
***KRAS***					0.652
	Wild-type	15	13	7.0 (3.2–10.8)	
	Mutant	77	64	6.0 (4.2–7.8)	
***TP53***					0.510
	Wild-type	40	33	5.0 (2.6–7.4)	
	Mutant	52	44	7.0 (5.0–9.0)	

Abbreviations: CI, confidence interval; PFS, progression-free survival.

**Table 5:** Multivariable Cox regression analysis for overall survival according to *KRAS* mutation status.

Variables	HR (95% CI)	*p*-Value
Age (continuous, per year)	1.01 (0.99–1.04)	0.349
ECOG ≥ 2 vs. 0–1	4.39 (2.04–9.42)	<0.001
De novo metastatic vs. recurrent	0.84 (0.43–1.65)	0.616
Primary tumor location, head vs. non-head	0.72 (0.40–1.29)	0.266
Metastatic sites (≥2 vs. <2)	1.37 (0.82–2.30)	0.230
FOLFIRINOX vs. non-FOLFIRINOX	1.05 (0.48–2.32)	0.902
*KRAS* mutant vs. wild-type	1.13 (0.56–2.28)	0.727

**Table 6:** Multivariable Cox regression analysis for progression-free survival according to *KRAS* mutation status.

Variables	HR (95% CI)	*p*-Value
Age (continuous, per year)	1.00 (0.97–1.02)	0.848
ECOG ≥ 2 vs. 0–1	3.26 (1.55–6.87)	0.002
De novo metastatic vs. recurrent	0.64 (0.36–1.15)	0.135
Primary tumor location, head vs. non-head	0.73 (0.42–1.26)	0.264
Metastatic sites (≥2 vs. <2)	1.08 (0.67–1.73)	0.756
FOLFIRINOX vs. non-FOLFIRINOX	0.98 (0.50–1.95)	0.961
*KRAS* mutant vs. wild-type	1.09 (0.59–2.01)	0.780

## Data Availability

The data presented in this study are available on request from the corresponding author due to institutional data protection policies.

## Supplementary Materials

The following supporting information can be downloaded at: https://www.mdpi.com/article/10.3390/curroncol33040216/s1, Table S1: Full list of molecular alterations detected by NGS; Table S2: Patient-level classification of non-*KRAS* alterations by clinical actionability; Table S3: Pathway-based grouping of potentially actionable alterations; Table S4: Multivariable Cox regression analysis—Overall survival (OS); Table S5: Multivariable Cox regression analysis—Progression-free survival (PFS).
