# Supplementary material for: Molecular Profiling and Survival Outcomes in Pancreatic Ductal Adenocarcinoma: A Multicenter Real-World Study from Turkey
Source: Curr Oncol. 2026 Apr 15;33(4):216. doi: 10.3390/curroncol33040216 (PMC13114962; doi:10.3390/curroncol33040216)
Supplement: Supplementary file 1 [file curroncol-33-00216-s001.zip › curroncol-4224498-supplementary.pdf]

Table S1. Full list of molecular alterations detected by NGS (N = 98).

Gene / Alteration	n	%
**KRAS**	82	83.7
**TP53**	58	59.2
**CDKN2A**	11	11.2
**SMAD4**	5	5.1
**ATM**	4	4.1
**ARID1A**	3	3.1
**BRCA2**	3	3.1
**GNAS8**	3	3.1
**SF3B1**	3	3.1
**FANCA**	2	2.0
**FBXW7**	2	2.0
**MCL1**	2	2.0
**STK11**	2	2.0
**AKT2 amp**	1	1.0
**AMER1**	1	1.0
**ARID2**	1	1.0
**ATR**	1	1.0
**AURKC**	1	1.0
**BAP1**	1	1.0
**BARD1**	1	1.0
**CCND1 amp**	1	1.0
**CCND2 amp**	1	1.0
**CCNE1 amp**	1	1.0
**CDK6 amp**	1	1.0
**CTNNB1**	1	1.0
**DPYD mut**	1	1.0
**EMYS amp**	1	1.0
**ERBB2 amp**	1	1.0
**ERBB2 mut**	1	1.0
**ERCC2**	1	1.0
**EZH2 amp**	1	1.0
**FGF19 amp**	1	1.0
**FGF3 amp**	1	1.0
**FGF4 amp**	1	1.0
**GNAQ**	1	1.0
**KMT2**	1	1.0
**MET exon 14 skipping**	1	1.0
**MLH1**	1	1.0
**MRE11 amp**	1	1.0
**MTRR**	1	1.0
**MUYTH**	1	1.0
**MYC amp**	1	1.0
**NF1**	1	1.0
**NOTCH4**	1	1.0
**NTRK2**	1	1.0
**PBRM1**	1	1.0
**PIK3CA**	1	1.0
**PMS1**	1	1.0
**RASA2**	1	1.0
**RNF43**	1	1.0
**ROS-1**	1	1.0
**SETD2**	1	1.0
**TGFBR2**	1	1.0
**TSC1**	1	1.0

Abbreviations: NGS, next-generation sequencing.

Table S2. Patient-level classification of non-KRAS alterations by clinical actionability

Clinical actionability category	Operational definition used in this study	Representative alterations identified in this cohort	n (%) of patients*
**Clinically actionable**	Alterations associated with an approved targeted therapy in a tumor-agnostic setting or with strong clinically established therapeutic relevance	**BRCA2 mutation**, **ROS1 fusion**	**4 (4.1)**
**Potentially actionable / investigational**	Alterations with emerging clinical evidence, off-label therapeutic implications, or potential relevance for clinical trial enrollment	**ATM mutation**, **CDKN2A alteration**, **STK11 mutation**, **ERBB2 amplification/mutation**, **PIK3CA mutation**, **MET exon 14 skipping**, **NF1 mutation**, **TSC1 mutation**, **AKT2 amplification**, **RASA2 mutation**, **RNF43 mutation**, **CDK6 amplification**, **CCND1/CCND2 amplification**, **CCNE1 amplification**, **ATR mutation**, **BARD1 mutation**, **ERCC2 mutation**, **FANCA mutation**, **MRE11 amplification**	**32 (32.7)**
**Non-actionable / prognostic only**	Alterations without established therapeutic relevance in routine PDAC management, or alterations considered biologically informative without current treatment implications	**TP53 mutation**, **SMAD4 mutation**, other non-targetable alterations, or cases with only KRAS alterations	**62 (63.3)**

Footnotes: * Percentages were calculated using the full cohort denominator (**N = 98**) and are **patient-based**, not alteration-based. Patients harboring more than one alteration were assigned to the **highest level of actionability** identified. Clinical actionability was classified using a **modified ESCAT-informed framework** adapted to PDAC and tumor-agnostic approvals. KRAS alterations were excluded from this table because, although biologically central in PDAC, they were not considered clinically actionable in routine practice for this cohort.

Table S3. Pathway-based grouping of potentially actionable alterations (N=98)

Therapeutic pathway	Associated alterations	n	%
CDK / Cell Cycle Control	CDKN2A, CDK6, CCND1/2, CCNE1	15	15.3
PARP / DNA Damage	ATM, BRCA2, BARD1, FANCA, ERCC2, ATR,	13	13.3

Therapeutic pathway	Associated alterations	n	%
Repair (DDR)	MRE11		
RTK / HER2 Signaling	ERBB2, MET exon 14 skipping, ROS1, NTRK2	5	5.1
mTOR / AKT Signaling	AKT2, PIK3CA, TSC1, STK11	5	5.1
MAPK / RAF / MEK	NF1, RASA2 (excluding KRAS)	2	2.0
WNT Signaling	RNF43	1	1.0

Footnote: Percentages are based on the full cohort (N=98). Alterations are grouped by putative targetable pathway; a patient may contribute to more than one pathway.

Table S4. Multivariable Cox regression analysis for overall survival according to TP53 mutation status

Variables	HR (95% CI)	p-value
**Age (continuous, per year)**	1.02 (0.99-1.05)	0.280
**ECOG ≥2 vs. 0-1**	4.57 (2.12-9.84)	<0.001
**De novo metastatic vs. recurrent**	0.85 (0.43-1.69)	0.644
**Primary tumor location, head vs. non-head**	0.74 (0.42-1.33)	0.318
**Metastatic sites (≥2 vs. <2)**	1.35 (0.81-2.26)	0.253
**FOLFIRINOX vs. non-FOLFIRINOX**	1.13 (0.50-2.55)	0.774
**TP53 mutant vs. wild-type**	1.32 (0.78-2.23)	0.309

Table S5. Multivariable Cox regression analysis for progression-free survival according to TP53 mutation status

Variables	HR (95% CI)	p-value
**Age (continuous, per year)**	1.00 (0.97-1.02)	0.873
**ECOG ≥2 vs. 0-1**	3.30 (1.56-6.96)	0.002
**De novo metastatic vs. recurrent**	0.64 (0.36-1.16)	0.141
**Primary tumor location, head vs. non-head**	0.74 (0.43-1.28)	0.282
**Metastatic sites (≥2 vs. <2)**	1.06 (0.66-1.70)	0.796
**FOLFIRINOX vs. non-FOLFIRINOX**	0.99 (0.50-1.99)	0.989
**TP53 mutant vs. wild-type**	1.13 (0.70-1.82)	0.616
